# Exploratory analysis of predictors of revision surgery for proximal junctional kyphosis or additional postoperative vertebral fracture following adult spinal deformity surgery in elderly patients: a retrospective cohort study

**DOI:** 10.1186/s13018-018-0960-5

**Published:** 2018-10-12

**Authors:** Hiroshi Uei, Yasuaki Tokuhashi, Masafumi Maseda, Masahiro Nakahashi, Hirokatsu Sawada, Koji Matsumoto, Hiroyuki Miyakata

**Affiliations:** Department of Orthopaedic Surgery, Nihon University School of Medicine, 30-1 Oyaguchi Kami-cho, Itabashi-ku, Tokyo, 173-8610 Japan

**Keywords:** Mean bone density, adult spinal deformity, Pedicle screw loosening, Proximal junctional kyphosis, Upper instrumented vertebra

## Abstract

**Background:**

Proximal junctional kyphosis (PJK) following adult spinal deformity (ASD) surgery in elderly patients is markedly influenced by osteoporosis causing additional vertebral fracture and loosening of pedicle screws (PS). This study aimed to investigate the association between mean bone density represented in Hounsfield units (HU) on spinal computed tomography (CT) and revision surgery for PJK or postoperative additional vertebral fracture following ASD surgery in elderly patients.

**Methods:**

The subjects were 54 ASD patients aged 65 years or older who were treated with correction and fusion surgery of four or more levels and could be followed for 2 years or longer. Bone density was measured before surgery using lumbar dual-energy X-ray absorptiometry (DXA) and spinal CT in all patients. The patients were divided into group A (*n* = 14) in which revision surgery was required for PJK or additional vertebral fracture and group B (*n* = 40) in which revision surgery was not required. We retrospectively investigated incidences of PJK, additional vertebral fracture, and PS loosening, perioperative parameters, radiographic parameters before and after surgery, and osteoporosis treatment administration rate.

**Results:**

No significant difference was noted in young adult mean (YAM) on DXA between groups A and B, respectively (*P* = 0.62), but the mean bone densities represented in HU of the T8 (*P* = 0.002) and T9 (*P* = 0.01) vertebral bodies on spinal CT were significantly lower in group A, whereas those of the L4 (*P* = 0.002) and L5 (*P* = 0.01) vertebral bodies were significantly higher in group A. The incidence of PJK was not significantly different (*P* = 0.07), but the incidence of additional vertebral fracture was significantly higher in group A (*P* < 0.001). The incidences of uppermost PS loosening within 3 months after surgery were 71% and 40% in groups A and B, respectively (*P* = 0.04).

**Conclusions:**

In elderly patients who required revision surgery, the mean bone densities of vertebral bodies at T8 and T9 were significantly lower. The mean bone density represented in HU on spinal CT may be useful for risk assessment of and countermeasures against revision surgery after ASD surgery in elderly patients.

## Background

The rates of revision surgery due to proximal junctional kyphosis/failure (PJK/PJF) following correction and fusion surgery for adult spinal deformity (ASD) is high, and its risk assessment and countermeasures are necessary but still insufficient [1–7]. The potentially modifiable risk factors are greater curvature correction, combined anterior-posterior spinal fusion, hybrid instrumentation (proximal hooks and distal pedicle screws), fusion to the sacro-pelvis, thoracoplasty procedure, and residual sagittal imbalance [4, 5, 7]. Non-modifiable risk factors include older age (> 55 years) and severe preoperative sagittal imbalance [4, 5, 7]. Other less well-established but likely risk factors of PJK/PJF following ASD surgery are low bone density, high body mass index, and presence of a comorbidity [4, 5], and the risk of revision surgery due to complications associated with not only PJK/PJF due to adjacent segment disease but also additional remote level vertebral fracture following posterior instrumentation fusion surgery has been a concern for elderly patients with low vertebral bone density [8, 9]. Bone density has been normally evaluated in an anteroposterior projection of the lumbar vertebra using dual-energy X-ray absorptiometry (DXA), but there are some problems with lumbar DXA in ASD patients. It is unclear whether it reflects the bone density of the lower thoracic vertebrae, in which upper instrumented vertebra (UIV) of ASD surgery is frequently present. Besides, no study in ASD patients on the bone density in the vertebrae around the UIV has been reported. The use of spinal CT substituting for lumbar DXA to complement this disadvantage has been reported, and a significant positive correlation between the *T*-score on DXA and Hounsfield units (HU) on spinal CT was reported [10]. Bone density on spinal CT can be measured at any vertebral level [10–13], and it can be investigated separately in the vertebral body and pedicle. In addition, accurate measurement is possible even in the presence of spinal deformity. Given the risk for possible neurological damage as well as severe back pain or impaired quality of life, PJK/PJF or additional remote level vertebral fracture is particularly serious complications for ASD patients. The etiology of PJK/PJF is multifactorial as no study has evaluated a single factor that strongly and consistently predicts their development. Furthermore, there remains conflicting evidence with regard to whether the number of levels fused, the UIV implant types, or the location of the UIV influence the risk of PJK/PJF development. Thus, we aimed to identify factors associated with revision surgery following correction and fusion surgery for ASD in elderly patients. Demographics, clinical data, and radiographic variables were analyzed.

## Methods

### Patient population

This study was a retrospective review of a prospectively collected data from 1654 consecutive patients who underwent spine surgery at our institution from January 2007 to December 2014. Ninety-six out of 1654 patients underwent ASD surgery. The inclusion criteria for the study were ASD patients aged 65 years or older who were treated with correction and fusion surgery of four or more levels and could be followed by our institution for 2 years or longer. We performed ASD surgery without the use of bone morphogenetic protein because it was not permitted by the Ministry of Health, Labour and Welfare in Japan. Regarding rod type, we used 6.0 titanium alloy dual rods from the same company for all cases. Patients with a past medical history of malignant cancer, Parkinson’s disease, secondary osteoporosis, metabolic bone disease other than osteoporosis, or those taking medications such as chronic glucocorticoids that cause a decrease in bone strength were excluded. This decision was made prior to study initiation by both two surgeons (H.U. and Y.T.). Seventy-three patients were included in this study, and 19 out of 73 patients were excluded. Twelve patients were lost to follow up, 2 patients had malignant cancer, 2 patients had Parkinson’s disease, 2 patients had secondary osteoporosis, and 1 patient had rheumatoid arthritis. The 54 patients were divided into two groups and retrospectively investigated: group A (*n* = 14) which required revision surgery due to severe pain, neurological deficits, or progressive sagittal deformity associated with PJK or additional vertebral fracture and group B (*n* = 40) which did not require revision surgery. Revision surgeries were performed for PJK in 12 and additional remote level vertebral fracture in 2. Postoperative follow-up was performed at 1, 3, 6, 9, and 12 months and every 6 months thereafter.

### Evaluation of bone density

**Fig. 1:**
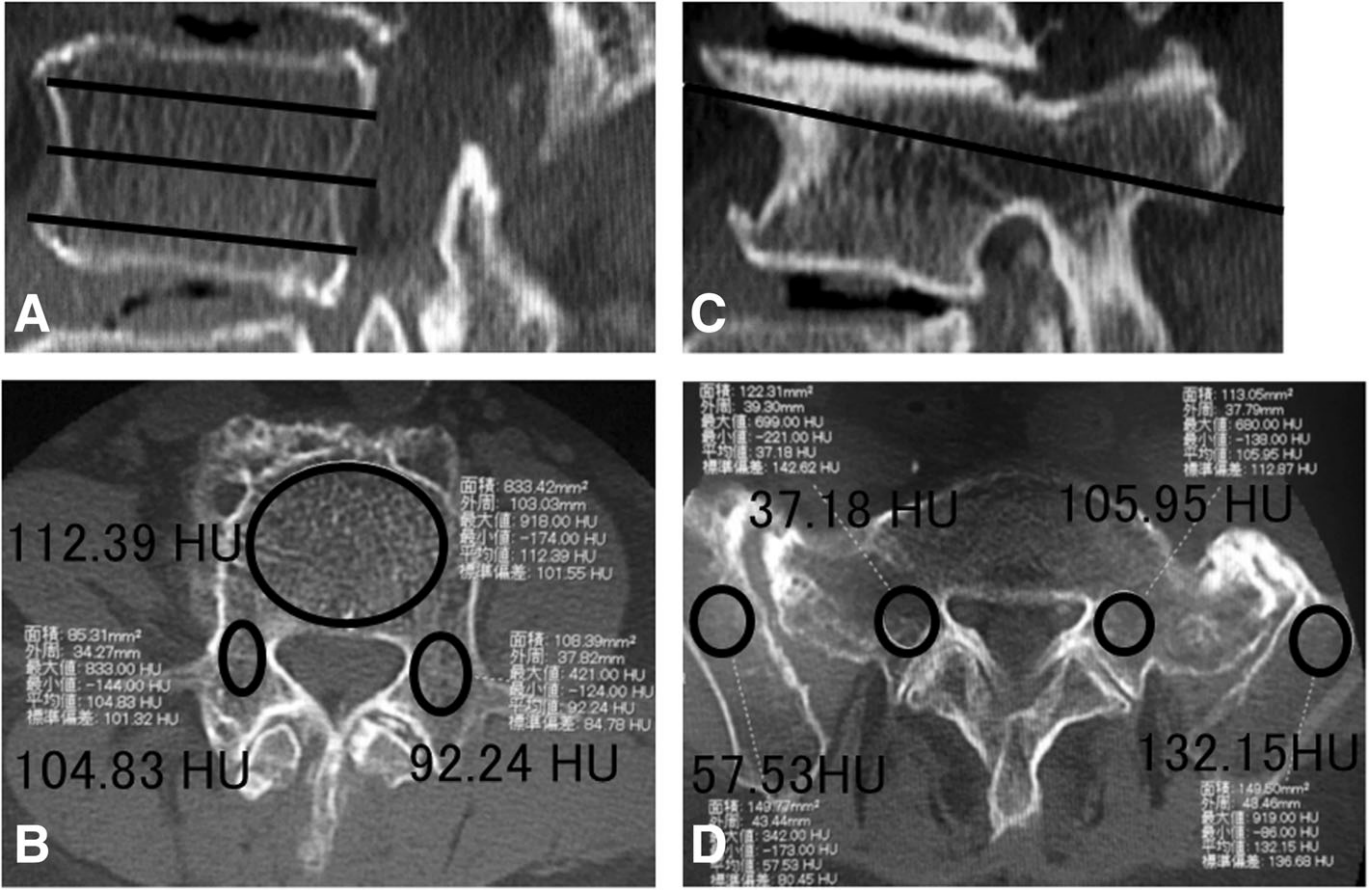
Bone density evaluation on spinal CT. CT images of the third lumbar and sacral vertebrae and ilium. The vertebral body was equally divided into three parts in the cranio-caudal direction (**a**), and the bone density was measured in the cancellous bone surrounded by an oval contour excluding the vertebral endplate and cortical bone (112.39 HU) (**b**). The bone density of the pedicle was measured on the bilateral sides excluding the cortical bone (right: 101.32 HU, left: 92.24 HU) (**b**) in the axial section at the center in the cranio-caudal direction of the pedicle in a parasagittal section on CT (**c**). In the ilium, the bone density was measured in an area similar to that in the S1 pedicle excluding the cortical bone in an axial section at the S1 vertebral level (right: 57.53 HU, left: 132.15 HU) (**d**). The bone density of the S1 pedicle was measured in the same section (right: 37.18 HU, left: 105.95 HU) (**d**)

We performed both lumbar DXA and spinal CT as a standard practice on ASD patients within 3 months before surgery. To evaluate bone density on lumbar DXA, the mean values in an anteroposterior projection of the L2, L3, and L4 vertebral bodies were calculated and evaluated based on the young adult mean (YAM) values. In the evaluation of bone density represented in HU on spinal CT, the middle lower thoracic over the lumbosacral vertebrae and pelvis were imaged in all patients. The images were digitized, and the bone density was calculated using the software SYNAPSE Enterprise-PACS (FUJIFILM Corporation, Tokyo, Japan), employing the method reported by Schreiber et al. [10]. The vertebral body was divided into one third cranial, one third central, and one third caudal regions excluding the vertebral endplate in the sagittal section (Fig. 1a), and the bone density of cancellous bone excluding cortical bone was measured in an oval pattern (Fig. 1b, d). The bone density of pedicle was similarly measured on the bilateral sides excluding cortical bone (Fig. 1b, d) in the axial section at the center in the cranio-caudal direction of the pedicle in a parasagittal section on spinal CT (Fig. 1c). In the ilium, the bone density was measured in the region excluding cortical bone in the axial section at the S1 level (Fig. 1c). The mean values of each measured levels represented in HU were calculated in the vertebral body, pedicle and ilium, and regarded as the mean bone densities at the level.

### Data collection

The demographic, clinical, and radiographic data collected included preoperative factors, perioperative factors, and postoperative factors. As preoperative factors, age, sex, body mass index (BMI), bone density (DXA and spinal CT), radiographic parameters, and presence or absence of preoperative treatment of osteoporosis were investigated. In bone density evaluation on spinal CT, the mean bone density was determined in T8, T9, T10, T11, T12, L1, L2, L3, L4, L5, S1, and ilium. The following radiographic parameters were measured: (1) sagittal vertical axis (SVA), the distance between the C7 plumb line and the posterosuperior corner of S1; (2) pelvic tilt (PT), the angle between the line connecting the midpoint of the sacral endplate to the middle axis of the femoral heads and the vertical; (3) thoracic kyphosis (TK), the angle between the upper endplate of T5 vertebra and the lower endplate of T12; (4) lumbar lordosis (LL), the angle between the lower endplate of T12 and the upper endplate of S1; (5) pelvic incidence (PI), the angle between the line perpendicular to the sacral endplate at its midpoint and the line connecting the point to the middle axis of the femoral heads; (6) UIV + 2 angle, the angle between the caudal endplate of the upper instrumented vertebra and the cranial endplate of the two supra-adjacent vertebra; and (7) PI-LL. On the basis of the above radiographic parameters, patients were additionally stratified by the SRS-Schwab ASD classification [14].

As perioperative factors, operation time, intraoperative blood loss, number of levels fused, implant types of UIV, level of uppermost PS, and presence or absence of pedicle subtraction osteotomy (PSO) and sacral fusion were investigated, and SVA, PT, TK, LL, change in LL (Postoperative LL–preoperative LL), PI, PI-LL, and UIV + 2 angle were investigated as radiographic parameters immediately after surgery. We distinguished the UIV levels from uppermost pedicle screw or hook. For example, when we placed bilateral pedicle screws at UIV level of T10, the uppermost pedicle screw level was T10. When we placed bilateral hooks at UIV level of T10, the uppermost pedicle screw level was T11. When we placed unilateral pedicle screw and unilateral hook at UIV level of T10, the uppermost pedicle screw level was T10.

As postoperative factors, duration of follow-up, incidences of additional vertebral fracture, PJK, and PS loosening, and treatment of osteoporosis were investigated. PJK was defined following the method reported by Glattes et al. [2]. The following conditions were regarded as PJK: (1) when the sagittal Cobb angle (UIV + 2 angle) formed by the caudal endplate of UIV and cephalad endplate of two vertebrae proximal (UIV + 2) was 10° or larger and (2) the UIV + 2 angle increased at least 10° greater than the preoperative measurement. Regarding PS loosening, the presence of a 1 mm or larger circumferential radiolucent zone around PS on plain radiographs acquired in two or more directions was judged as PS loosening [15].

Statistical analysis was performed using the SPSS 19.0 version software (SPSS Inc., Chicago, IL, USA). The chi-square test for independence was used for the nominal scales, the *t* test or Mann-Whitney *U* test was used for the data scales, and the significance level was set at 5%.

## Results

**Fig. 2:**
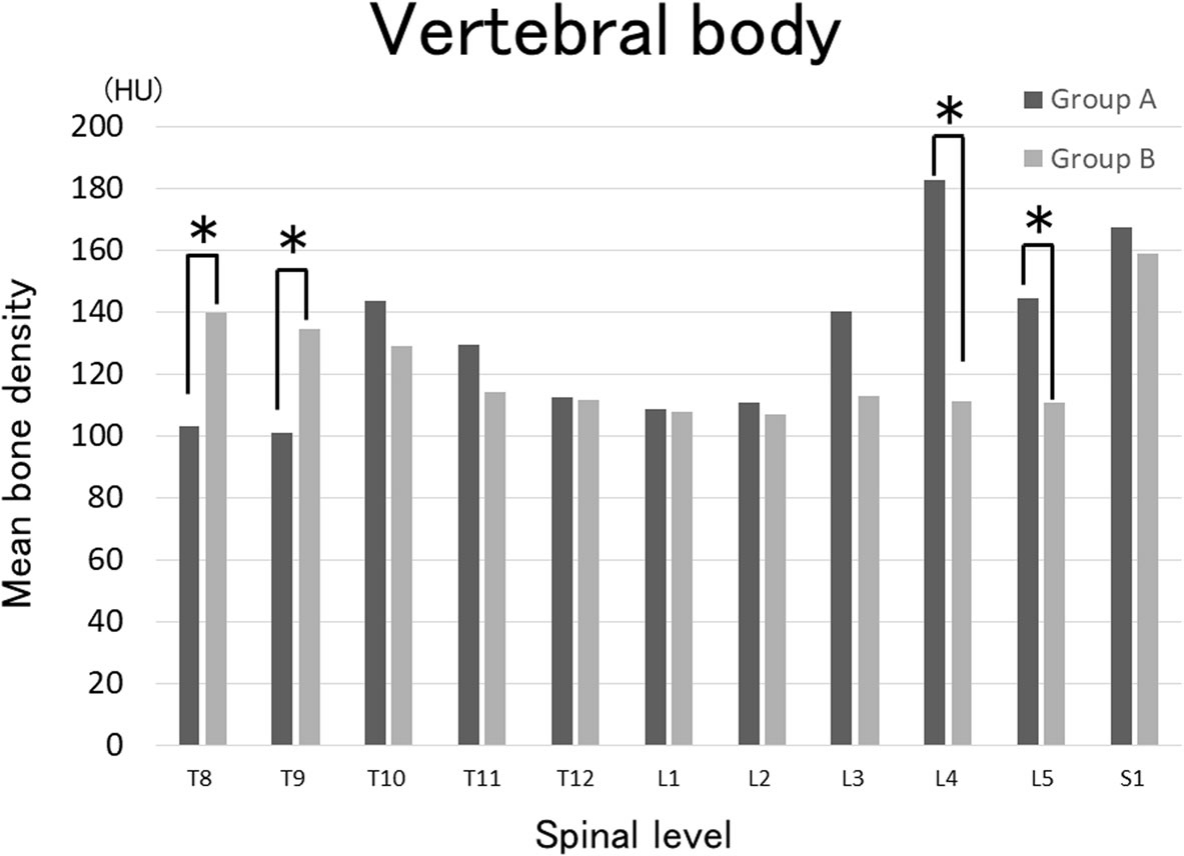
Evaluation of mean bone density of the vertebral bodies on spinal CT. The mean bone densities of T8 were 103 and 139 HU in group A and B, respectively (*P* = 0.002), and those of T9 were 101 and 134 HU, respectively (*P* = 0.01). In contrast, those of L4 were 182 and 111 HU, respectively (*P* = 0.002) and those of L5 were 144 and 111 HU, respectively (*P* = 0.01), being significantly higher in group A. *Significant difference between groups with *P* < 0.05

**Fig. 3:**
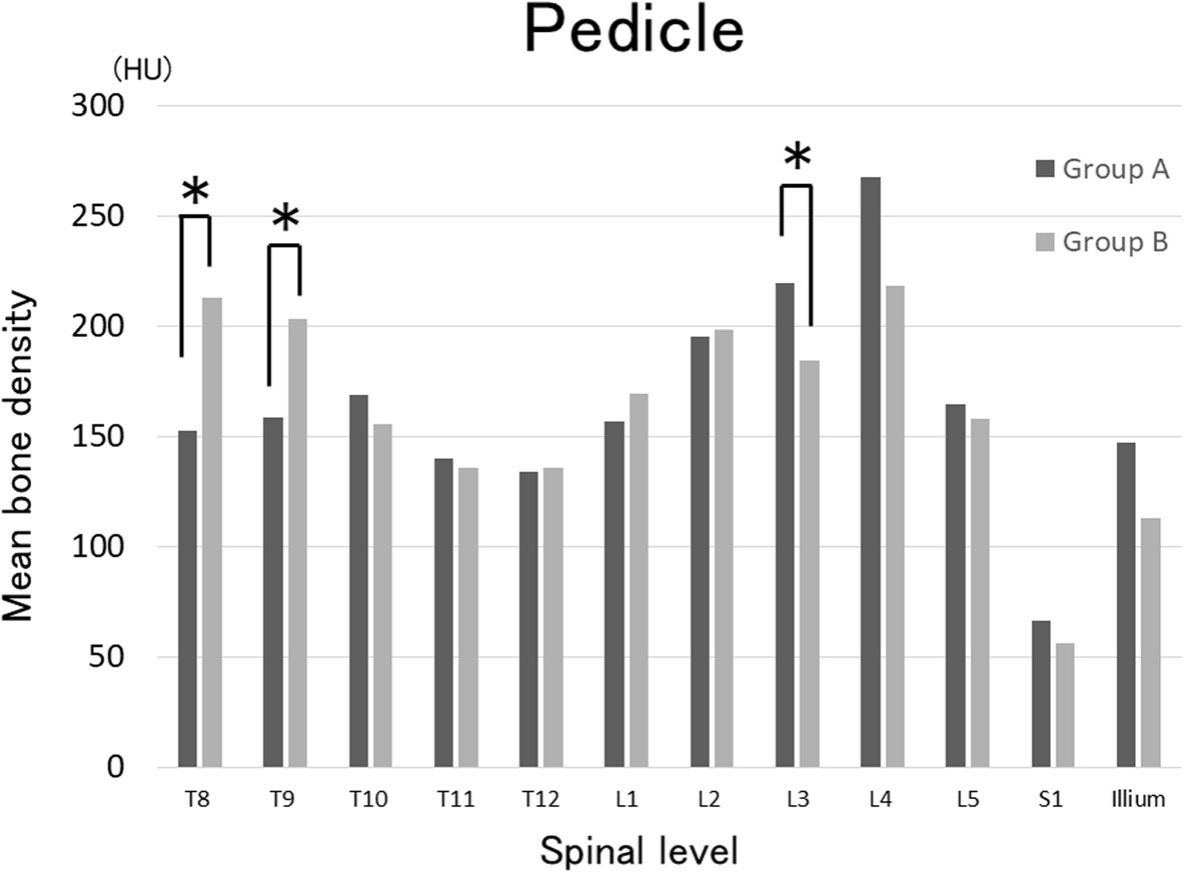
Evaluation of mean bone density of the pedicle on spinal CT. The mean bone densities of the T8 pedicle were 158 and 213 HU in group A and B, respectively (*P* = 0.03) and those of the T9 pedicle were 158 and 203 HU, respectively (*P* = 0.02), being significantly higher in group B. In contrast, those of the L3 pedicle were 219 and 184 HU, respectively, being significantly higher in group A (*P* = 0.04). *Significant difference between groups with *P* < 0.05

Mean age at the time of surgery was 73.0 and 74.2 years old, respectively (*P* = 0.55) (Table 1). Groups A and B included 14 (100%) and 36 (90%) female patients, respectively (*P* = 0.28). Group B had a greater mean BMI (*P* = 0.01). Indication for the index surgical procedure included degenerative scoliosis in 4 and 12 patients, degenerative kyphosis in 4 and 8, degenerative kyphoscoliosis in 2 and 8, and posttraumatic kyphosis in 2 and 10, in groups A and B respectively, and posttraumatic kyphoscoliosis in 2 in group B, and iatrogenic kyphosis in 2 in groups A (*P* = 0.18). The mean YAM value on DXA was 92.9% and 96.2%, respectively (*P* = 0.62). The number of patients with osteoporosis or osteopenia evaluated on lumbar DXA were 6 (42.9%) and 18 (45%), respectively (*P* = 1.0). The mean bone densities of T8 (*P* = 0.002) and T9 (*P* = 0.01) on spinal CT were significantly lower in group A (Fig. 2), whereas those of L4 (*P* = 0.002) and L5 (*P* = 0.01) were significantly higher in group A. The mean bone densities of the T8 (*P* = 0.03) and T9 (*P* = 0.02) pedicles were significantly lower in group A (Fig. 3), whereas that of the L3 pedicle (*P* = 0.04) was significantly higher in group A. Preoperative treatment of osteoporosis was performed in 6 (42.8%) and 22 patients (55%), respectively (*P* = 0.54). Regarding the radiographic parameters before surgery, no significant difference was noted in SVA, PT, TK, LL, PI, PI-LL, or UIV + 2 angle between the groups. The proportions of the SRS-Schwab ASD classification were not also significantly different between the groups (Table 2).

**Table 1:** Baseline characteristics in group A and group B

Characteristic	Group A (*n* = 14)	Group B (*n* = 40)	*P* value
Age at surgery, mean (SD), years	73.0 (5.5)	74.2 (6.0)	0.55
Female, *n* (%)	14 (100)	36 (90)	0.28
BMI, mean (SD), kg/m^2^	20.5 (3.6)	23.6 (4.0)	0.01
Diagnosis, *n* (%)			0.18
Degenerative scoliosis	4 (28.6)	12 (30)	
Degenerative kyphosis	4 (28.6)	8 (20)	
Degenerative kyphoscoliosis	2 (14.3)	8 (20)	
Posttraumatic kyphosis	2 (14.3)	10 (25)	
Posttraumatic kyphoscoliosis	0	2 (5)	
Iatrogenic kyphosis	2 (14.3)	0	
BMD YAM, mean (SD), %	92.9 (21.6)	96.2 (21.0)	0.62
Osteopenia or osteoporosis, *n* (%)	6 (42.8)	18 (45)	1.0
No. of preoperative treatment for osteoporosis, *n* (%) preoperative radiographic parameters	6 (42.8)	22 (55)	0.54
Sagittal vertical axis, mean (SD), mm	103.4 (57.0)	90.0 (48.6)	0.39
Pelvic tilt, mean (SD), degrees	30.3 (9.7)	27.7 (8.3)	0.33
Thoracic kyphosis, mean (SD), degrees	15.0 (12.9)	25.0 (17.4)	0.07
Lumbar lordosis, mean (SD), degrees	8.9 (15.0)	20.7 (16.1)	0.09
Pelvic incidence, mean (SD), degrees	47.0 (3.3)	50.1 (7.8)	0.21
PI-LL, mean (SD), degrees	38.1 (12.8)	29.5 (14.8)	0.14
UIV + 2 angle, mean (SD), degrees	7.4 (5.2)	3.7 (7.0)	0.11

**Table 2:** Baseline SRS-Schwab adult spinal deformity classification for group A and group B

	Group A (*n* = 14)	Group B (*n* = 40)	*P* value
Coronal curve type, *n* (%)			
T	0	0	0.37
L	4 (20)	8 (28.6)	
D	0	0	
N	10 (80)	32 (71.4)	
PI-LL, *n* (%)			
0	0	4 (10)	0.11
+	0	6 (15)	
++	14 (100)	30 (75)	
Global alignment, *n* (%)			
0	3 (21.4)	5 (12.5)	0.71
+	5 (35.7)	17 (42.5)	
++	6 (42.9)	18 (45)	
Pelvic tilt, *n* (%)			
0	2 (14.3)	7 (17.5)	0.74
+	5 (35.7)	10 (25)	
++	7 (50)	23 (57.5)	

PI-LL: 0, < 10°; +, 10–20°; ++, > 20°. Global alignment, based on C7 SVA value: 0, < 4 cm; +, 4–9.5 cm; ++, > 9.5 cm. Pelvic tilt: 0, < 20°; +, 20–30°; ++, > 30°

**Fig. 4:**
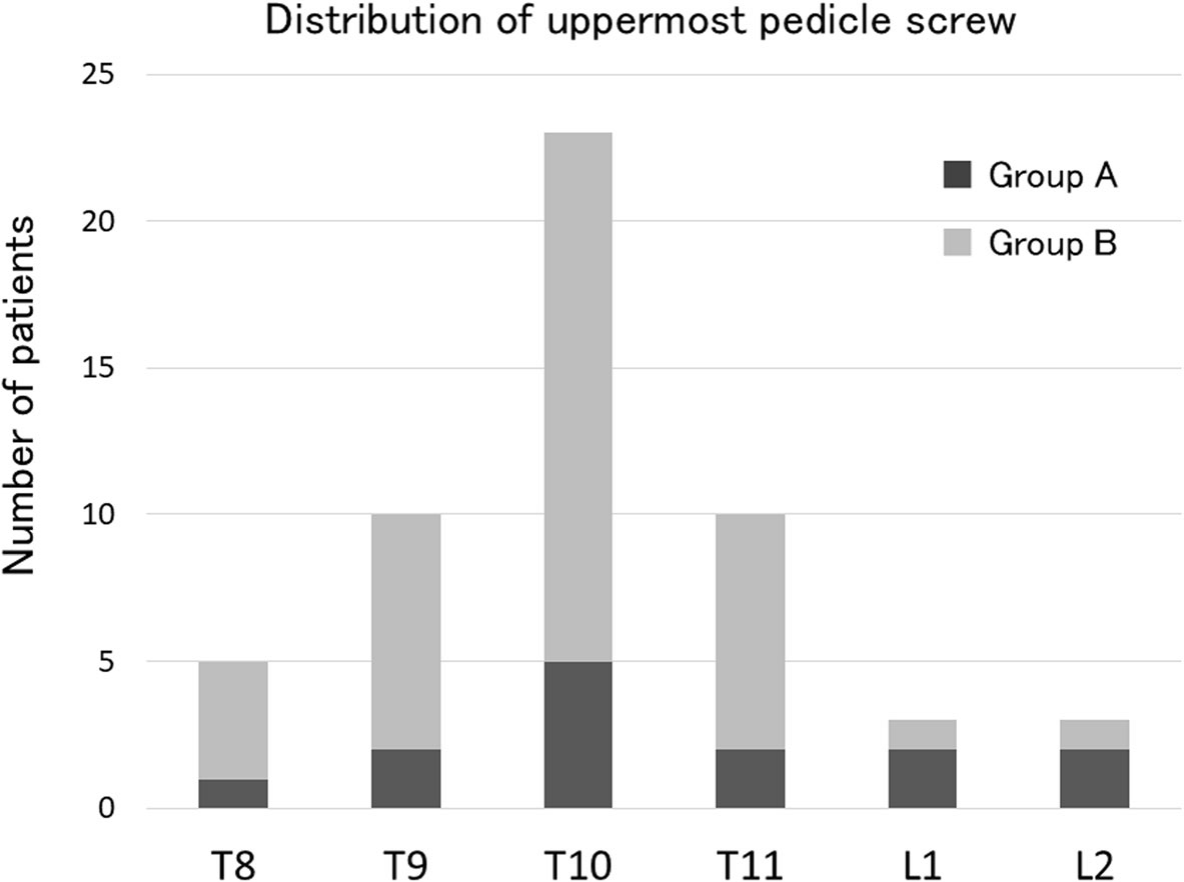
Level of uppermost PS. The most frequent level of uppermost PS was T10 in both groups without a significant difference between the 2 groups (*P* = 0.3)

Regarding the perioperative factors, mean operation time were 282 and 304 min in groups A and B, respectively (*P* = 0.37), mean blood losses were 698 and 1128 mL, respectively (*P* = 0.02), mean number of levels fused were 6.9 and 7.6, respectively (*P* = 0.24), and UIV implant type was PS in more than half of the patients (*P* = 0.30) (Table 3). The level of uppermost PS was not significantly different between the groups (*P* = 0.32) (Fig. 4). PSO was applied to 14% and 30% in groups A and B, respectively (*P* = 0.21), and sacral fusion was applied in 57% and 70%, respectively (*P* = 0.28). No significant difference was noted between the groups in any radiographic parameter immediately after surgery.

**Table 3:** Comparison of perioperative parameters in group A and group B

Parameter	Group A (*n* = 14)	Group B (*n* = 40)	*P* value
Operation time, mean (SD), min	282.1 (82.7)	304.7 (79.4)	0.37
Blood loss, mean (SD), mL	698.6 (335.1)	1128.0 (949.1)	0.02
No. of levels fused, mean (SD)	6.9 (2.7)	7.6 (1.7)	0.24
UIV implant types, *n* (%)			0.30
Pedicle screws	9 (64.3)	22 (55)	
Hooks	3 (21.4)	16 (40)	
Unilateral pedicle screw and unilateral hook	2 (14.3)	2 (5)	
No. of pedicle subtraction osteotomy, *n* (%)	2 (14.2)	12 (30)	0.21
No. of fusion to sacrum, *n* (%)	8 (57.1)	28 (70)	0.28
Immediate postoperative radiographic parameters			
Sagittal vertical axis, mean (SD), (mm)	70.2 (29.6)	63.2 (24.8)	0.45
Pelvic tilt, mean (SD), (degrees)	30.2 (9.9)	25.4 (7.6)	0.08
Thoracic kyphosis, mean (SD), (degrees)	31.4 (16.8)	31.4 (13.4)	0.66
Lumbar lordosis, mean (SD), (degrees)	26.0 (11.7)	28.7 (11.3)	1.0
Change in LL, mean (SD), degrees	17.1 (17.0)	10.0 (13.0)	0.11
Pelvic incidence, mean (SD), degrees	46.7 (2.9)	49.5 (7.8)	0.32
PI-LL, mean (SD), (degrees)	22.2 (13.6)	21.9 (7.8)	0.94
UIV + 2 angle (SD), degrees	8.9 (5.6)	7.7 (7.9)	0.52

Regarding the postoperative factors, the mean durations of follow-up after surgery were 40 and 37 months in groups A and B, respectively (*P* = 0.61) (Table 4), additional vertebral fracture developed in 85% and 25%, respectively (*P* < 0.001), PJK developed in 85% and 60%, respectively (*P* = 0.07), and PS loosening occurred in 100% and 95%, respectively (*P* = 0.54). As for the PJK levels in revision surgeries, there were 1 case at T7, 2 cases at T8, 5 cases at T9, 2 cases at T10, and 2 cases at T12, respectively. Loosening of the uppermost PS occurred in 100% and 75%, respectively (*P* = 0.04), and it occurred within 3 months after surgery in 71% and 40%, respectively (*P* = 0.04). Postoperative treatment of osteoporosis was performed in 100% and 77%, respectively (*P* = 0.04), and 14% and 40% of them were treated with teriparatide, respectively (*P* = 0.07).

**Table 4:** Comparison of postoperative clinical results in group A and group B

Parameter	Group A (*n* = 14)	Group B (*n* = 40)	*P* value
Follow up, mean (SD), (months)	40.4 (19.5)	37.4 (19.3)	0.61
No. of postoperative fracture, *n* (%)	12 (85.7)	10 (25)	< 0.001
No. of proximal junctional kyphosis, *n* (%)	12 (85.7)	24 (60)	0.07
No. of PS loosening, *n* (%)	14 (100)	38 (95)	0.54
No. of uppermost PS loosening, *n* (%)	14 (100)	30 (75)	0.04
No. of uppermost PS loosening within 3 months, *n* (%)	10 (71.4)	16 (40)	0.04
No. of lowermost PS loosening, *n* (%)	10 (71.4)	32 (80)	0.37
No. of postoperative treatment for osteoporosis, *n* (%)	14 (100)	34 (77.2)	0.04
No. of teriparatide use, *n* (%)	2 (14.2)	16 (40)	0.07

## Discussion

In this study, patients who required revision surgery due to complications associated with PJK or additional remote level vertebral fracture after ASD surgery had significantly lower mean bone densities of T8 and T9 vertebra and significantly higher mean bone densities of L4 and L5 vertebra. The rates of loosening of the uppermost PS within 3 months after surgery and additional vertebral fracture were significantly higher in patients who required revision surgery.

Since the sagittal alignment of the spine is often corrected largely in ASD surgery and this loads a large physical stress on the adjacent intervertebral segments, PJK is likely to develop [5, 7, 16]. PJK caused by bone failure and implant/bone interface failure are strongly influenced by the bone strength of UIV and nearby vertebrae. Bone strength is generally evaluated based on the bone density. Bredow et al. measured the mean bone density of vertebrae on spinal CT in 365 patients aged 59 years on average treated with PS fixation of one or two levels and observed that PS loosening occurred in the vertebrae with a mean bone density of 116 HU, but it did not occur in those with a mean bone density of 132 HU [12]. Kumano et al. reported that lower BMD assessed using HU values from preoperative CT is associated with adjacent segment fracture after spinal fusion surgery [8]. The present study clarified that in patients who required revision surgery due to complications associated with PJK or additional remote level vertebral fracture after ASD surgery, PS loosening occurred early and the mean bone density was low in the T8 and T9 vertebral bodies and high in the L4 and L5 vertebral bodies. For these patients, certain countermeasures may be necessary, such as strengthening of osteoporosis treatment and augmentation of PS.

The incidence of uppermost PS loosening was significantly higher in group A, and it occurred within 3 months after surgery in 71% and 40% in groups A and B, respectively, being significantly higher in group A. PS loosening does not necessarily cause PJK or additional vertebral fracture, but it was clarified that the probability of revision surgery is high when uppermost PS loosening occurs within 3 months after surgery. To prevent uppermost PS loosening early after surgery, risk assessment and certain countermeasures are necessary. It has been reported that the threshold of the mean bone density of the vertebra for applying augmentation to prevent PS loosening is about 120 HU [12]. In our study, the most frequent level of the uppermost PS was T10 and the mean bone densities of the vertebral body were 143 and 129 HU in groups A and B, respectively, being not significantly different between the groups, and both values were higher than 120 HU. However, the mean bone densities of the T8 and T9 vertebral bodies were about 100 HU in group A whereas these were about 140 HU in group B, being significantly different. Besides, there were more than half of the patients whose PJK levels were at T8 (2cases) and T9 (5cases) out of the 12 cases in group A. These results clarified that PJK should be strongly influenced by the bone density of nearby UIV. Also, mean bone densities of the T8 and T9 vertebral bodies whose uppermost PS levels except for T9 and T10 were 107 and 109 HU in group A, respectively, being significantly low. It was suggested that the risk of uppermost PS loosening early after ASD surgery is high in elderly patients regardless of the level of the uppermost PS when the mean bone densities of the T8 and T9 vertebral bodies are lower than about 140 HU.

Ohtori et al. reported the effect of drugs for treatment of osteoporosis on PS loosening [17]: PS loosening occurred in 26% and 25% of patients on CT in the oral risedronate treatment and control groups at 12 months after surgery, respectively, whereas the incidence was 13% in the teriparatide injection group, being significantly lower. In our study, no significant difference was noted in the incidence of PS loosening between patients with and without preoperative treatment of osteoporosis, and on the contrary, fewer patients received postoperative treatment of osteoporosis in group B. However, limited to teriparatide, the drug was more frequently used in group B, although the difference was not significant. To prevent uppermost PS loosening early after surgery, pre- and postoperative treatment with teriparatide or certain PS augmentation such as polymethylmethacrylate cement injection and/or expandable PS may be necessary [1, 6, 18].

The limitations of this study were the retrospective design, multiple factors associated with revision surgery to be tested, and small number of patients who were included in the study. In the baseline characteristics of SRS-Schwab ASD classification, there were more kyphosis patients in group A and more scoliosis patients in group B. Kyphosis patients tend to have highly degenerated lumbar vertebra, and sclerosed changes could occur in curved vertebra in scoliosis patients. Patients in group A could have more sclerosed lumbar vertebra, and patients in group B could have more sclerosed thoracic vertebra. The higher mean bone density of L4 and L5 in group A and T8 and T9 in group B could be caused by the sclerosed vertebra. Therefore, if we performed a matched pair analysis in each curve type, results could be different from the current study. However, baseline characteristics excluding BMI, preoperative parameters, and postoperative parameters excluding blood loss were not statistically different between the groups. Second, there were some biases in deciding to perform additional surgery. There were many patients who developed PJK in both group A (85%) and group B (60%) in the current study. Clinical symptoms of patients with PJK/PJF were variable, and patients who needed revision surgery could have not decided to have additional surgery. However, patients who received revision surgery had severe symptoms such as severe pain, neurological deficits, or progressive sagittal deformity. Thus, there were no patients with mild symptoms included in group A who needed not have revision surgery. Another limitation was the diversity of UIV implant types, such as bilateral PS, bilateral hooks, and unilateral PS with unilateral hook, and this made the risk assessment of PS loosening based on the mean bone density of UIV difficult. Therefore, we compared the mean bone density of the extensive region from T8 to the ilium. Another limitation was the slightly broad range of the UIV level from lower thoracic to upper lumbar vertebrae. We considered that the inclusion of patients with UIV at an upper lumbar level is more practical because no significant bias was noted in the basic background between the groups.

## Conclusions

We focused on the factors associated with revision surgery for proximal junctional kyphosis or additional postoperative vertebral fracture following correction and fusion surgery for ASD in elderly patients. In patients who required revision surgery, the mean bone densities of vertebral bodies and pedicles at T8 and T9 were significantly lower. The mean bone density represented in HU on spinal CT may be useful for risk assessment of and countermeasures against revision surgery after ASD surgery in elderly patients.

## Abbreviations

ASD: Adult spinal deformity
BMI: Body mass index
CT: Computed tomography
DXA: Dual-energy X-ray absorptiometry
HU: Hounsfield units
LL: Lumbar lordosis
PI: Pelvic incidence
PJF: Proximal junctional failure
PJK: Proximal junctional kyphosis
PS: Pedicle screw
PSO: Pedicle subtraction osteotomy
PT: Pelvic tilt
SVA: Sagittal vertical axis
TK: Thoracic kyphosis
UIV: Upper instrumented vertebra
YAM: Young adult mean
